# Artificial Intelligence in the Design and Development of Nanoparticle Drug Delivery Systems: A Systematic Review

**DOI:** 10.1155/adpp/5531134

**Published:** 2026-09-21

**Authors:** Razan Haddad

**Affiliations:** ^1^ Department of Pharmaceutical Sciences, Faculty of Pharmacy, Jadara University, Irbid 21110, Jordan, jadara.edu.jo

**Keywords:** artificial intelligence, drug delivery, machine learning, nanomedicine, nanoparticles, predictive modeling

## Abstract

**Background:**

Artificial intelligence (AI) has rapidly emerged as a powerful tool for accelerating the design and optimization of nanoparticle‐based drug delivery systems (NDDSs). By analyzing complex multidimensional datasets, AI models can predict nanoparticle physicochemical properties, biodistribution, and safety profiles more efficiently than traditional experimental approaches.

**Methods:**

This systematic review was conducted following PRISMA 2020 guidelines and was registered in PROSPERO (ID: 1148160). Five electronic databases (PubMed, Scopus, Web of Science, ScienceDirect, and SpringerLink) were searched up to January 2025. Studies applying AI approaches, including machine learning, deep learning, and hybrid modeling to nanoparticle design, optimization, biodistribution prediction, or nanotoxicology, were included. Extracted data included nanoparticle platform, AI methodology, prediction targets, validation strategies, and reported performance metrics. Methodological quality was assessed using a framework integrating TRIPOD‐AI and Joanna Briggs Institute (JBI) appraisal domains.

**Results:**

A total of 17 unique studies met the established eligibility criteria and were included in the final qualitative analysis. The applications of machine learning or deep‐learning methods and models were demonstrated across the various nanoparticle‐based platforms to predict the formulation properties, biodistribution, nano–bio interactions, therapeutic materials discovery, and nanoparticle toxicity. The models augmented by artificial intelligence (AI) technology in the physiologically based pharmacokinetic area manifested promising prediction ability for biodistribution, while the abilities differed among the datasets and the prediction endpoints. Nine studies incorporated in vitro or in vivo biological/experimental validation, whereas only four of the 17 included studies used independent external validation.

**Conclusions:**

AI has significant potential to enhance nanoparticle formulation optimization, biodistribution modeling, and toxicity prediction. Nevertheless, translation into clinical practice remains constrained by small datasets, limited external validation, and inconsistent reporting standards. Future research should prioritize standardized datasets, transparent AI models, and prospective validation to advance AI‐enabled nanomedicine toward clinical implementation.

## 1. Introduction

The rapidly growing applications of artificial intelligence (AI) in drug formulation and nanomedicine make it a revolutionary aid in this part of the pharmaceutical industry. Nanoparticle drug delivery systems (NDDSs) offer several advantages such as improved solubility, increased duration in circulation, controlled release, and enhanced targeting of drugs toward their sites of action [1–6]. Nevertheless, although significant progress in the field of engineering nanoparticles has been achieved, their successful application in therapy is limited since only liposomal or polymer‐based formulations have been approved for therapeutic use [7–10].

A significant obstacle in developing nanomedicine is the complication of the nano–bio interface, which implies that a huge number of physicochemical characteristics of a compound such as size, shape, weight, zeta potential, surface chemistry, density of ligands, forming protein corona, loading efficiency of drugs, and kinetics of release affect biodistribution, immune recognition, cellular uptake, and efficacy [10–16]. It has been established that slight changes in the surface properties or chemical composition of nanoparticles may change the macrophage uptake, endothelial transport, and clearance pathways [17–21]. As a result, conventional trial‐and‐error approaches to formulation development are rather slow, expensive, and unrepeatable, thus making the NDDS development process time and resource‐consuming [22–25].

In addition to formulation improvement, drug design is an important part of pharmaceutical development because the molecular and physicochemical properties of a drug candidate affect biological effect, pharmacokinetics, and the applicability of the drug in drug delivery systems. Recent advances in drug design and nanomaterial‐based delivery methods have increasingly combined drug design processes with data‐driven practices, high‐throughput experiments, automation, and computational methods, providing a basis for developing better, more customizable treatment systems [26].

This merger forms a solid basis for the increased use of AI and machine learning in research in the area of drug delivery based on nanoparticles. The utilization of AI‐enabled computation methods is increasingly put into practice in the solutions to the abovementioned issues. The existing applications in the pharmaceutical and nanomedicine research area comprise machine‐learning methods, deep‐learning methods, and hybrid modeling techniques aimed at revealing complicated nonlinear relationships under nanomaterial properties, formulation characteristics, and biological results [27–32]. Compared to that, some more sophisticated approaches including reinforcement learning and graph neural networks appear merely to be topics for further research rather than established applications in the reference materials processed in the considered systematic review.

The first uses of AI methods in computational pharmaceutics included quantitative structure–activity relationship (QSAR) models, physiologically based pharmacokinetics (PBPK), predictive toxicology, and formulation development, laying the groundwork for the development of data‐based pharmaceutical design and predictive formulation improvement [33–36]. These studies demonstrated the application of algorithms, particularly support vector machines, random forests (RFs), and artificial neural networks (ANNs), to predict nanoparticle size, encapsulation efficiency, biodistribution, and toxicity. AI‐supported PBPK technologies allowed for predicting tumor accumulation and in vivo distribution of nanoparticles, with variable predictive performance across datasets and endpoints. Moreover, new perspectives underline the growing influence of AI‐supported projects in various fields of pharmaceutical and food‐related technologies [37–39].

Based on these previous developments, new advancements (2023–2025) have highlighted expanding and emerging applications of AI in nanomedicine, including biodistribution modeling, nanotoxicology, and protein‐corona prediction, as well as prospective applications in adaptive dosing optimization and individualized treatment design. These latter emerging applications provide broader context for future developments in the field and were not represented as established applications among the 17 primary studies included in the present systematic review. Deep‐learning techniques such as convolutional neural networks (CNNs) and recurrent networks (RNNs) have been explored for predicting in the simulation of biological barrier violation and release kinetics; moreover, GNNs have been explored for predicting interactions between nanoparticles and proteins and their targeting characteristics [40–43]. In addition, reinforcement learning technologies have been studied for solving problems related to adaptive dose scheduling and sequential synthesis optimization. Furthermore, explainable AI (XAI) approaches such as SHapley Additive exPlanations (SHAP) and Local Interpretable Model‐agnostic Explanations (LIME) have made progress in ensuring the explanation of the phenomena behind the toxicity and targeting aspects of nanoparticles [6, 44–47].

**FIGURE 1:**
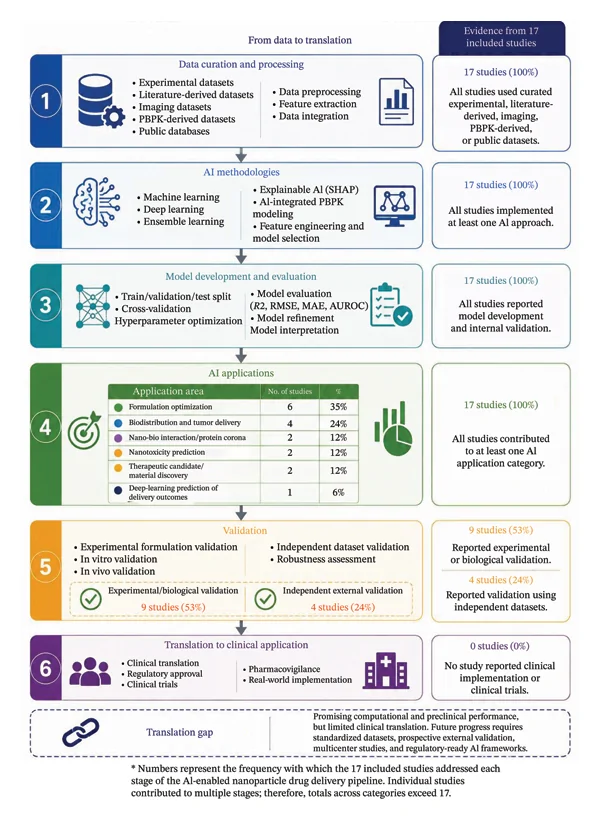
AI workflow for nanoparticle delivery system design and development, illustrating the process from data curation to AI methodologies (machine learning, deep learning, and hybrid modeling) to applications in formulation optimization, nano–bio interaction prediction, biodistribution modeling, toxicity prediction, and therapeutic material discovery.

However, there are several significant translation barriers that exist. The majority of studies lack coherence, use few data, have inconsistent reporting practices, and many of them only rely on internal validation, which leads to a lack of replicability and generalization. What is more, nontransparent deep‐learning technologies are widely criticized for their lack of transparency and comprehensibility, as well as for problems with their potential regulation [48–56] (Figure 1).

Multiple recent systematic studies have looked at the role played by AI in nanomedicine, particularly looking at the various algorithms used, formulation improvement, and aspects of nanotoxicology and other advanced technologies [31, 57–62]. For instance, advanced versions of systematic reviews have successfully analyzed the use of AI in the development of nanoparticles and predictive nanotoxicology, but most of these analyses have concentrated on discussing the performance of the algorithms rather than how well they have been validated, how they were validated, and how ready they are for clinical application [63, 64]. This study improves upon the earlier systematic reviews by engaging methodological quality assessment, biological validation, external validation, and readiness for translation in a single Preferred Reporting Items for Systematic Reviews and Meta‐Analyses (PRISMA) 2020–based framework for all four aspects of the process. The quality of methodology was assessed through the complementary use of Joanna Briggs Institute (JBI) and TRIPOD‐AI appraisal domains, leading to a more thorough assessment of methodological rigor with respect to clinical application readiness.

With AI applications emerging quickly in the area of NDDSs, a comprehensive synthesis of evidence is needed to assess current methodological strengths, biological implications of results, existing limitations, and future possibilities.

Thus, this systematic review has the following aims:

1. To analyze the situation with AI methods used in nanoparticle formulation, improvement, delivery, and toxicity studies.
2. To examine the biological validation strategies applicable to the studies included in this review.
3. To appraise the current maturity of AI applications and their readiness for practical use.
4. To identify the main problems and future research directions to advance nanomedicine into practice.

## 2. Methodology

This review followed PRISMA 2020 guidance to promote transparency and reproducibility in the identification, screening, eligibility assessment, and inclusion of studies. A comprehensive search of PubMed, Scopus, Web of Science, ScienceDirect, and SpringerLink encompassed literature published up to January 2025. Search strings combined controlled vocabulary and free‐text terms related to “artificial intelligence,” “machine learning,” “deep learning,” “reinforcement learning,” “predictive modeling,” “nanoparticles,” “nanomedicine,” and “drug delivery.” Reference lists of retrieved articles were also scanned to capture additional records [65].

Refinement and verification of the search process were performed by adding supplementary keywords related to AI and nanomedicine to the original search word. The process included the utilization of keywords such as “graph neural networks,” “nanoarchitectonics,” and “PBPK modeling.” The newly found studies were contrasted with the results obtained through the primary search strategy to discover the number of additional studies revealed through new keywords. While further research allowed the retrieval of some additional literature, the analysis of the material revealed that no unique studies were retrieved through the use of added terms. The process was used for evaluating the thoroughness of the last search strategy; however, it was not regarded as the formal sensitivity testing of the evidence synthesis. The various databases utilized in this review (PubMed, Scopus, Web of Science, ScienceDirect, and SpringerLink) represent a very broad cross section of the biomedical literature but may not include some of the highest quality journals or articles published more recently that are not indexed by these databases. To reduce this caveat to the search results, the reference lists of included studies were examined manually to identify and locate additional relevant publications. However, this could also lead to potential biases by introducing selection biases. Inclusion criteria were peer‐reviewed English‐language studies that applied AI techniques to the design, characterization, optimization, or evaluation of NDDSs and reported outcomes for formulation performance, targeting, toxicity prediction, pharmacokinetics, or therapeutic efficacy. In order to guarantee consistent and reliable evaluation of the eligibility of full text as well as data extraction in line with the language capabilities of the review team, the search was confined to publications in the English language. For each eligible study, data were extracted on nanoparticle platform, AI methodology, prediction target, validation strategy (e.g., internal cross‐validation, independent test set, external validation, in vitro validation, or in vivo validation), primary biological endpoint, and reported AI performance metrics. The selection of these extraction domains was informed by recent recommendations for transparent reporting and evaluation of AI‐enabled biomedical research [66–68]. The author and an independent researcher screened titles, abstracts, and full‐text articles according to the predefined eligibility criteria. Disagreements were resolved through discussion and consensus, with consultation from a senior researcher when necessary. Although agreement between reviewers was reviewed retrospectively from the screening records to ensure consistency, formal inter‐rater reliability statistics were not prospectively planned and are therefore not reported.

**FIGURE 2:**
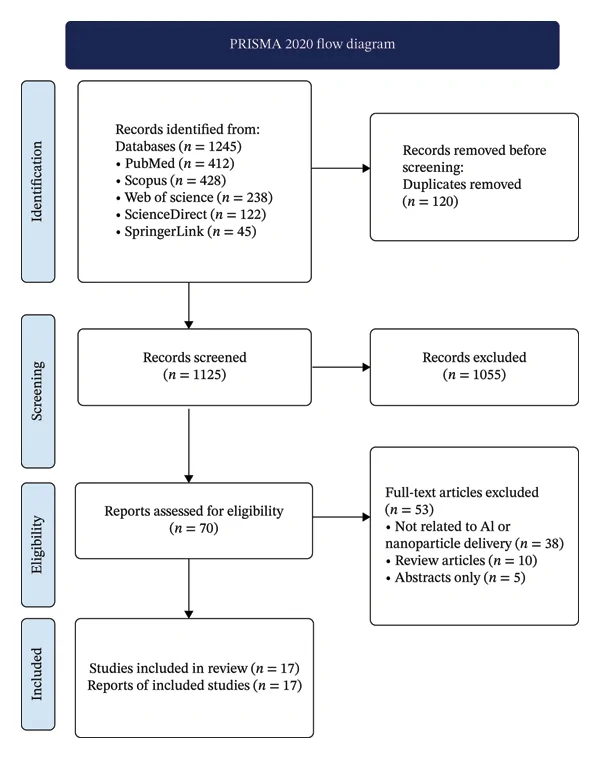
PRISMA 2020 flow diagram illustrating study selection process for the systematic review (*n* = 17 included studies).

The review protocol was prospectively registered in PROSPERO (Registration ID: 1148160) before the completion of study selection. The review was conducted according to the registered protocol. No substantive methodological amendments affecting the eligibility criteria, search strategy, outcomes, or quality assessment framework were made after registration (Figure 2).

This review is not without limitations. Despite systematic searching, heterogeneity in datasets, study designs, and outcome reporting constrained the comparability of findings. Moreover, the limitation of eligibility to publications in the English language can prevent some relevant studies that had been published in other languages from being selected, which can lead to the incompleteness of the evidence base and the appearance of language bias. Additionally, many of the included studies used small datasets, raising the risk of overfitting. The evolving role of the protein corona and its impact on biological fate remains incompletely modeled, although recent mechanistic studies offer valuable guidance [69–71]. Another challenge is the absence of standardized benchmarks and open‐access datasets, which impedes reproducibility, although FAIR data initiatives are emerging to address this gap [72, 73]. Beyond technical concerns, regulatory frameworks are still in early stages, with both the U.S. Food and Drug Administration (FDA) and European Medicines Agency (EMA) emphasizing the need for transparency and ethical safeguards [74, 75]. Future directions must prioritize integration of multiomics data, development of federated and collaborative models, and incorporation of XAI methods, as highlighted in recent position papers [76–78]. Addressing these limitations will be critical to accelerating the safe and effective translation of AI‐enabled nanomedicine.

### 2.1. Quality Assessment and Risk of Bias

Methodological quality and risk of bias were assessed using a structured appraisal framework integrating selected domains from the TRIPOD‐AI reporting guideline (model development transparency, predictor selection, dataset description, internal and external validation, overfitting mitigation strategies, calibration and discrimination reporting, and reproducibility) and relevant domains from the JBI Critical Appraisal Checklist for analytical cross‐sectional and experimental studies (selection bias, confounding, outcome measurement validity, and statistical appropriateness).

A predefined scoring rubric was developed to operationalize the combined framework. Each domain was rated as “Yes” (1 point), “Partial” (0.5 points), or “No” (0 points). The detailed operational definitions used for assigning “Yes”, “Partial”, and “No” for each appraisal domain are provided in Supporting Table S2 to facilitate reproducibility. The maximum achievable score was 15 points. Studies were categorized as follows:

• High methodological quality: ≥ 12 points.
• Moderate quality: 8–11.5 points.
• Low quality: < 8 points.

The methodological quality assessment was conducted independently by the author and an independent researcher using the predefined appraisal framework. Any discrepancies were resolved through discussion and consensus. Quality scores were used descriptively to identify common methodological limitations and were not used to exclude studies from the evidence synthesis. This systematic review was conducted and reported in accordance with the PRISMA 2020 Statement. A completed PRISMA 2020 checklist is provided as Supporting File.

## 3. Results

Within the domain of formulation optimization, neural networks and hybrid models have been applied to predict polymeric nanoparticle characteristics and drug encapsulation efficiencies [23, 79–81]. Similarly, PBPK and multiscale modeling studies demonstrate how biodistribution can be forecasted using AI approaches [82, 83]. In toxicity prediction, gradient boosting, ensemble models, and extreme learning machines have been applied to nanotoxicology datasets, underscoring the importance of predictive safety frameworks [84–87]. In addition to the 17 main research studies presented in this review, the broader literature has discussed emerging applications of AI in personalized nanomedicine, like the creation of dosage forms and treatment response monitoring [81, 88–90]. These possible applications offer crucial background for understanding the emerging trends of AI‐aided nanomedicine; however, it must not be considered as the results obtained by studies included in this article.

### 3.1. Overview of Included Studies

In total, 17 primary studies met the eligibility criteria defined in the study. These studies correspond to Refs. [91–107] and are separate from the other references mentioned in other sections of the paper, which are included to provide context and commentary. All 17 primary studies reported in Tables 1 and 2 allow for a precise cross‐reference between the results of studies reported in the PRISMA flow diagram.

**TABLE 1:** Characteristics of the included studies (*n* = 17).

No.	Study	NP platform	Application	AI model	Dataset	Inputs	Outputs	Validation	Main finding
1	Ban et al. [91]	Multiple nanoparticles; protein corona	Nano–bio interaction/cellular recognition	Machine learning regression/classification	Integrated nanoparticle–protein‐corona dataset	Nanoparticle physicochemical and biological‐environment descriptors	Functional corona composition and cellular recognition	Comparison with experimental proteomic and cellular recognition data	Predicted functional protein‐corona composition and linked it to cellular recognition.
2	Yan et al. [92]	Nanostructure image datasets	Nano–bio interaction prediction	Convolutional neural network with image interpretation	Nanostructure images linked with experimental nano–bio endpoints	Image‐derived nanostructure features	Nano–bio interaction endpoints	Cross‐validation against experimental endpoint data	Demonstrated that CNN‐derived image features can predict nano–bio interactions.
3	Harrison et al. [93]	Lipid nanoparticles	Early prediction of LNP delivery outcomes	Deep‐learning models	Time‐lapse microscopy of LNP‐treated cells	Early microscopy/image features	Later delivery/expression outcomes	Train/validation/test analysis using experimental microscopy data	Showed that early microscopy signals can predict later LNP delivery outcomes.
4	Munteanu et al. [94]	Drug‐decorated nanoparticles	Antiglioblastoma candidate prioritization	Perturbation‐theory ML; bagging classifier	Drug–nanoparticle combinations represented by molecular and experimental descriptors	Drug, nanoparticle and assay descriptors	Antiglioblastoma activity	Training/test assessment and large virtual screening	Prioritized potentially active antiglioblastoma drug–nanoparticle combinations.
5	Wang et al. [95]	Electrosprayed micro‐/nanoparticles	Particle‐size prediction	Random forest, XGBoost and comparator models	Experimental electrospray formulation/process dataset	Formulation and process parameters	Particle size	Cross‐validation plus experimental testing	ML predicted electrospray particle size and identified influential process variables.
6	Lin et al. [96]	Multiple nanoparticle systems in tumor‐bearing mice	Tumor‐delivery prediction	DNN, RF, SVM, linear and bagged models	376 PBPK‐derived datasets	Nanoparticle, administration and tumor‐model variables	Tumor delivery at multiple time points	Training/test comparisons	AI‐PBPK integration was feasible, but predictability varied across endpoints and time points.
7	Dawoud et al. [97]	Lecithin/chitosan nanoparticles	Formulation optimization	ANN integrated with Quality by Design	Experimentally prepared formulations	Formulation factors	Particle size, PDI, entrapment, zeta potential and release	Experimental characterization, release and cytotoxicity testing	ANN‐QbD optimization identified a formulation with favorable critical quality attributes.
8	Hoseini et al. [98]	Liposomal nanoparticles	Size and PDI prediction	Ensemble machine learning/least‐squares boosting	Literature‐derived liposome formulation dataset	Formulation and process variables	Particle size and PDI	Training/test evaluation	Predicted liposome size and PDI from formulation/process factors.
9	Chou et al. [99]	Multiple nanoparticles in tumor‐bearing mice	Biodistribution and tumor‐delivery prediction	AI‐assisted PBPK model	288 nanoparticle PBPK datasets	Nanoparticle physicochemical and experimental variables	Biodistribution and tumor accumulation	Comparison with in vivo–derived datasets	AI‐assisted PBPK showed useful but heterogeneous predictive performance.
10	Ma et al. [100]	Tumor‐targeted nanoparticles	Tumor‐delivery efficiency prediction	XGBoost with SHAP	Nanoparticle physicochemical and tumor‐genomic dataset	Tumor‐genomic mutations and nanoparticle properties	Delivery efficiency	Cross‐validation with explainability analysis	Integrated tumor genomic and nanoparticle features and identified influential delivery predictors.
11	Ahmadi et al. [101]	Multiple nanoparticle types	Nanoparticle toxicity prediction	Comparative machine‐learning models	Compiled nanotoxicity dataset	Nanoparticle physicochemical and exposure descriptors	Toxicity class/response	Model comparison and internal validation	Compared ML approaches for predicting nanoparticle toxicity.
12	Chaurawal et al. [102]	Fucoidan/polyethyleneimine sorafenib nanoparticles	Anticancer formulation optimization	Machine learning with DoE‐ANN	Experimentally prepared self‐assembled nanoparticle formulations	Formulation‐factor levels	Critical quality attributes and anticancer performance	Experimental characterization and in vitro anticancer assessment	Integrated ML and DoE‐ANN to optimize sorafenib‐loaded self‐assembled nanoparticles.
13	Seegobin et al. [103]	PLGA nanoparticles	Production optimization	XGBoost and other ML models compared with DoE	Experimental nanoprecipitation dataset	Formulation and process variables	Particle size and zeta potential	Cross‐validation and comparison with experimental DoE models	Combining ML with DoE improved prediction and interpretation of PLGA nanoparticle production.
14	Maharjan et al. [104]	mRNA‐lipid nanoparticles	Multiattribute formulation/process optimization	XGBoost/Bayesian optimization and self‐validated ensemble model	Twenty‐four experimentally prepared formulations	Material ratios and microfluidic process attributes	Size, PDI, zeta potential, pKa, EE, recovery and encapsulated mRNA	Predictions compared with newly prepared formulations	The ensemble model closely predicted several mRNA‐LNP critical quality attributes.
15	Khokhlov et al. [105]	Multiple nanoparticle types	Dynamic toxicity prediction	Machine‐learning classification and regression models	In vitro and/or in vivo toxicity records	Nanoparticle descriptors, exposure time and experimental variables	Toxicity category and viability over time	Internal model comparison	Extended nanotoxicity prediction to time‐dependent toxicity and viability outcomes.
16	Li et al. [106]	Ionizable‐lipid mRNA LNPs	Ionizable‐lipid discovery	Machine learning plus combinatorial chemistry	584 synthesized lipids; virtual library of 40,000; top 16 tested	Ionizable‐lipid structural descriptors	mRNA transfection potency	Prospective synthesis and experimental in vitro/in vivo testing	Demonstrated prospective AI‐guided discovery of ionizable lipids for mRNA delivery.
17	Mahdi et al. [107]	Multiple nanoparticle systems in tumor‐bearing mice	Organ‐specific biodistribution and tumor‐delivery prediction	Bayesian ridge regression, kernel ridge regression and K‐nearest neighbors; RFE and Firefly optimization	534 nanoparticle biodistribution records	Nanoparticle type/material, targeting strategy, cancer/tumor model, shape, size, zeta potential and administered dose	Delivery efficiency in tumor, heart, liver, spleen, lung and kidney	Cross‐validated train/test analysis using a previously compiled in vivo–derived dataset	KRR generally provided the strongest organ‐specific predictions, although performance varied across organs.

**TABLE 2:** Study‐level quantitative performance and experimental/biological validation of the 17 primary studies included in the systematic review.

No.	Study	AI/model	Predicted/optimized outcome	Reported quantitative result	Validation
1	Ban et al. [91]	Machine‐learning regression/classification	Functional corona composition and cellular recognition	*R* ^2^ > 0.75 reported for protein‐corona prediction	Comparison with experimental proteomic and cellular recognition data
2	Yan et al. [92]	Convolutional neural network with image interpretation	Nano–bio interaction endpoints	Study‐specific *R* ^2^ values; verify exact endpoint‐level values in full text	Cross‐validation against experimental endpoint data
3	Harrison et al. [93]	Deep‐learning models	Later delivery/expression outcomes	No single comparable summary metric stated in the abstract	Train/validation/test analysis using experimental microscopy data
4	Munteanu et al. [94]	Perturbation‐theory ML; bagging classifier	Antiglioblastoma activity	AUROC 0.96; test accuracy about 87%	Training/test assessment and large virtual screening
5	Wang et al. [95]	Random forest, XGBoost and comparator models	Particle size	Experimental RMSE reported for XGBoost and RF; verify exact values in full text	Cross‐validation plus experimental testing
6	Lin et al. [96]	DNN, RF, SVM, linear, and bagged models	Tumor delivery at multiple time points	Test *R* ^2^ varied by time point, approximately 0.33–0.70	Training/test comparisons
7	Dawoud et al. [97]	ANN integrated with quality by design	Particle size, PDI, entrapment, zeta potential and release	Model‐specific ANN statistics require full‐text verification	Experimental characterization, release and cytotoxicity testing
8	Hoseini et al. [98]	Ensemble machine learning/least‐squares boosting	Particle size and PDI	MAE 1.652 for size and 0.0105 for PDI	Training/test evaluation
9	Chou et al. [99]	AI‐assisted PBPK model	Biodistribution and tumor accumulation	Adjusted *R* ^2^ ≥ 0.70 in 133/288 datasets; selected endpoints about 0.82–0.83	Comparison with in vivo–derived datasets
10	Ma et al. [100]	XGBoost with SHAP	Delivery efficiency	Exact model metrics require checking in the full article	Cross‐validation with explainability analysis
11	Ahmadi et al. [101]	Comparative machine‐learning models	Toxicity class/response	Exact model‐specific values require full‐text verification	Model comparison and internal validation
12	Chaurawal et al. [102]	Machine learning with DoE‐ANN	Critical quality attributes and anticancer performance	Exact ANN/DoE statistics require full‐text verification	Experimental characterization and in vitro anticancer assessment
13	Seegobin et al. [103]	XGBoost and other ML models compared with DoE	Particle size and zeta potential	XGBoost was the best‐performing model for particle‐size prediction; verify exact statistics in full text	Cross‐validation and comparison with experimental DoE models
14	Maharjan et al. [104]	XGBoost/Bayesian optimization and self‐validated ensemble model	Size, PDI, zeta potential, pKa, EE, recovery and encapsulated mRNA	Overall prediction > 94% for XGBoost/Bayesian and > 97% for the ensemble model	Predictions compared with newly prepared formulations
15	Khokhlov et al. [105]	Machine‐learning classification and regression models	Toxicity category and viability over time	Exact model‐level metrics require full‐text verification	Internal model comparison
16	Li et al. [106]	Machine learning plus combinatorial chemistry	mRNA transfection potency	Lipid 119‐23 outperformed benchmark lipids in multiple tissues	Prospective synthesis and experimental in vitro/in vivo testing
17	Mahdi et al. [107]	BRR, KRR, and KNN with RFE/Firefly tuning	Delivery efficiency in tumor, heart, liver, spleen, lung and kidney	KRR test *R* ^2^: tumor 0.703, heart 0.900, liver 0.791, spleen 0.964, lung 0.604 and kidney 0.964. Reported RMSE: 2.605, 0.631, 8.436, 0.8975, 0.354 and 0.896, respectively.	Cross‐validation and held‐out test analysis of 534 records; no prospective biological validation

*Note:* LNP, lipid nanoparticle; NP, nanoparticle; PBPK, physiologically based pharmacokinetic; PDI, polydispersity index; SHAP, SHapley Additive exPlanations.

Abbreviations: AI, artificial intelligence; ANN, artificial neural network; CNN, convolutional neural network; DNN, deep neural network; DoE, design of experiments; EE, encapsulation efficiency; MAE, mean absolute error; PTML, perturbation‐theory machine learning; RF, random forest; RMSE, root‐mean‐square error; SVM, support vector machine.

The 17 included studies encompassed a wide spectrum of AI methods and nanoparticle platforms, as shown in Table 1. However, most studies relied on internal validation procedures, such as cross‐validation or random training–test splits. Explicit validation using a completely independent external dataset was uncommon, with only four of the 17 included studies reporting external validation. This represents an important limitation of the existing evidence because internally validated models may overestimate predictive performance and may not generalize adequately to different formulations, experimental settings, or biological systems. Nine studies incorporated some form of experimental or biological assessment alongside AI modeling. Ban et al. evaluated predicted protein‐corona composition and nanoparticle cellular recognition using proteomic and cell‐based experiments, whereas Yan et al. compared image‐derived predictions with experimentally measured nano–bio interaction endpoints [91, 92]. Harrison and coworkers utilized experimental time‐lapse microscopy for training and evaluating deep‐learning models with lipid nanoparticle‐treated cells [93]. In a different study, Wang and coauthors conducted machine‐learning predictions and experimental assessments of electrosprayed particle size, while Dawoud and coworkers investigated the characteristics of lecithin/chitosan nanoparticle formulation and determined its drug release and cytotoxicity [95, 97]. The research of Chaurawal et al. included physicochemical characterization and in vitro anticancer assessment of AI‐assisted sorafenib nanoparticle formulation [102]. Seegobin and coauthors conducted a comparative study of predicted and machine‐made PLGA nanoparticles; Maharjan et al. created mRNA‐lipid nanoparticle formulations to investigate predicted critical quality attributes [103, 104]. Finally, Li et al. have synthesized the AI‐predicted ionizable lipids and tested them for mRNA delivery both in laboratory settings and in living organisms [106].

The included studies demonstrated the broad applicability of AI across nanoparticle drug delivery research. Six studies focused on formulation or manufacturing optimization and the prediction of critical nanoparticle attributes, including particle size, polydispersity index (PDI), zeta potential, encapsulation efficiency, recovery, and drug‐release characteristics. These teams relied on various machine‐learning approaches: ANNs, RFs, XGBoost, ensemble learning methods, least‐squares boosting, and Bayesian optimization [95, 97, 98, 102, 103].

Four research teams set out to explore how nanoparticles spread through the body and whether they effectively reach tumors [87, 99, 100, 107]. Lin and Chou’s groups combined machine learning with PBPK modeling, a way to simulate how drugs move through living systems, whereas Ma’s team developed an interpretable XGBoost–SHAP model that could actually show which nanoparticle and tumor features mattered most for delivery success.

Despite that, many of these investigations were retrospective and related to old laboratory‐derived models rather than innovative prospective validation on new groups of animals. Chou et al. obtained adjusted *R* ^2^ values equal to or higher than 0.70 for 133 out of 288 datasets, while selected tumor‐delivery accounting measures reached their values of around 0.82–0.83; thus, predictive capability can be described more accurately as heterogeneous than highly effective.

There were two investigations devoted to molecular interactions and protein‐corona behavior (Ban et al.; Yan et al.), two projects studied toxic effects of nanoparticles (Ahmadi et al.; Khokhlov et al.), and two other studies tackled the issue of discovering the potential therapy (Munteanu et al.; Li et al.) [91, 92, 94, 101, 105, 106]. Harrison et al. employed advanced analytical techniques for pre‐evaluating results of lipid nanoparticle delivery processes [93]. In the study of Ma et al., an XAI approach was used to perform SHAP analysis, while structural and optimization techniques interface had been implemented by researchers interested in finding specific ecology and bioparameters [100].

Mahdi et al. applied Bayesian ridge regression, kernel ridge regression, and K‐nearest neighbor models to forecast where nanoparticles end up both in tumors and in major organs [107].

Collectively, the evidence shows that AI has been implemented at every step in the nanoparticle development process from formulation optimization to nano–bio interaction modeling, toxicity evaluation, biodistribution forecasting, and therapeutic material discovery. The formulation optimization studies were statistically the most grounded, while biodistribution, toxicity, and tumor‐delivery studies tend to depend more on historical data sets and internal validation. Even if some studies reported consistency between modeling and experimental findings, the biological validation was mostly confined to small‐scale in vitro studies, formulation research, or preclinical animal testing. Thus, the results show feasibility and potential preclinical success, but do not confirm the possibility of application in real‐world practice.

### 3.2. Performance of AI‐Enabled Formulation Optimization

Optimization of formulations was found to be the dominant use of AI in the available studies, comprising almost one‐third of the studies selected for review. The reports used machine‐learning techniques and deep‐learning algorithms that allowed predicting and optimizing key quality attributes such as size, PDI, zeta potential, encapsulation effectiveness, drug release, and technology parameters. The major algorithms adopted in the research are ANNs, RFs, XGBoost, least‐squares boosting, ensemble learning, and Bayesian optimization [95, 97, 98, 102–104].

Wang and collaborators applied machine‐learning models, primarily XGBoost and RF to predict the particle size of microparticles and nanoparticles produced by the electro‐spraying process based on formulation and technological parameters used [95]. Hoseini and colleagues managed to create an ensemble machine‐learning model for size and PDI predictions of liposomes based on formulation and manufacturing parameters [98].

Optimizing using ANNs has also been integrated with quality by design (QbD). Dawoud et al. achieved optimization of lecithin/chitosan nanoparticles using ANNs. The resulting particle properties were validated using various characterization techniques [97]. Similarly, Chaurawal et al. integrated machine learning with design of experiments (DoE) and ANN modeling to come up with the optimized drug carrier formulations [102].

Other more recent studies also indicate the use of advanced machine‐learning algorithms in optimizing nanoparticles’ manufacturing. Seegobin et al. reported a comparison of XGBoost and regular DoE for PLGA nanoparticles production and found that machine learning gave better predictions for particle size [103]. Maharjan et al. used XGBoost, Bayesian optimization, and an ensemble learning framework for the optimization of the mRNA‐lipid nanoparticle [104].

The optimized models predicted multiple critical quality attributes, including particle size, PDI, zeta potential, pKa, encapsulation efficiency, recovery, and encapsulated mRNA, and these predictions were subsequently confirmed using newly prepared formulations.

Collectively, these studies demonstrate that AI‐assisted formulation optimization can efficiently model complex relationships between formulation composition, manufacturing parameters, and nanoparticle quality attributes across diverse nanoparticle platforms, including polymeric, lipid‐based, and hybrid systems. Rather than replacing experimental development, AI approaches facilitate the identification of promising formulation conditions, reduce unnecessary experimental iterations, and improve the efficiency of nanoparticle formulation development through data‐driven optimization [95, 97, 98, 102–104].

### 3.3. Modeling Biodistribution and Targeting

AI has been put into practice more often to forecast how nanoparticles disperse in the body and target tumors by using techniques like machine learning and PBPK modeling. Four included studies focused on predicting nanoparticle biodistribution or tumor delivery using existing experimental data and computational modeling approaches [96, 99, 100, 107].

Lin et al. implemented various machine‐learning models such as deep learning, RFs, support vector machines, and bagging methods along with the usage of PBPK models for the purpose of predicting how effectively nanoparticles would deliver at various time points. The outcome revealed that predictive performances of various models differ and the test *R* ^2^ scores fluctuated in the range of around 0.33 to 0.70 [96]. In a related study, Chou et al. created a machine‐learning–assisted PBPK methodology based on 288 datasets of nanoparticles to make predictions about the accumulation of nanoparticles in tumors and their distribution in the organism. The authors obtained adjusted *R* ^2^ values of 0.70 or higher for 133 datasets, while the selected tumor drug delivery endpoints demonstrated *R* ^2^ values of around 0.82–0.83. Nevertheless, it should be noted that this level of accuracy was obtained for less than 50% of the datasets under analysis, which shows that predictive ability is highly variable in terms of different nanoparticle systems. This means that while indeed machine‐learning–based PBPK can assist in predicting the accumulation in tumor and effectiveness of drug delivery, the performance of the models is primarily dependent on properties of nanoparticles, biological endpoints used, and the composition of the dataset used [99].

In addition, Ma et al. created an interpretable model XGBoost with the use of SHAP to predict the effectiveness of drug delivery in terms of the physical and chemical properties of the nanoparticles as well as the genomic characteristics of the tumor. The study demonstrated the potential of XAI by determining the contribution of each variable to the predicted model effectiveness [100].

More recently, Mahdi et al. have employed different forms of algorithms such as Bayesian ridge regression, kernel ridge regression, and K‐nearest neighbor to forecast the efficiency of drug delivery from nanoparticles in different organs including the tumor, liver, spleen, kidneys, heart, and lungs using 534 biodistribution records [107].

Overall, kernel ridge regression brought satisfying results but with differing levels of prediction efficiency in organ‐specific cases, which illustrates the challenges of applying AI models in various biological systems. In general, the findings suggest that AI‐assisted biodistribution modeling has potential to enhance nanoparticle delivery efficiency modeling, yet the majority of studies in the field used retrospective data and internal validation while not applying external validation in its prospective manner at all. Therefore, we can conclude that the existing evidence is insufficient to consider the practices tested as actually generalizable. The studies above vary significantly in terms of the types of nanoparticles used, the aims of predicting, the data used for validation, and different assessment techniques employed (e.g., *R* ^2^, RMSE, MAE, AUROC, accuracy), and therefore, quantitative combination and formal meta‐analysis were not possible. Thus, the results are summarized using qualitative synthesis.

Table 3 summarizes the methodological characteristics of the included studies, whereas Table 2 provides study‐level quantitative performance metrics where these were reported.

**TABLE 3:** Synthesis of AI applications, validation, and evidence maturity.

Application category	Studies	No.	Main AI methods	Main outcomes	Validation basis	Interpretation
Nano–bio interaction and protein corona	Ban et al. [91]; Yan et al. [92]	2	CNN and classical ML	Corona composition, cellular recognition and nano–bio endpoints	Experimental endpoint data	Mechanistically valuable; external validation is limited.
Formulation and process optimization	Wang et al. [95]; Dawoud et al. [97]; Hoseini et al. [98]; Chaurawal et al. [102]; Seegobin et al. [103]; Maharjan et al. [104]	6	ANN, ensembles, RF, XGBoost, Bayesian optimization	Particle size, PDI, zeta potential, EE, release and process quality	Mostly direct experimental formulation data	Most mature and experimentally grounded application category.
Biodistribution and tumor delivery	Lin et al. [96]; Chou et al. [99]; Ma et al. [100]; Mahdi et al. [107]	4	AI‐PBPK, XGBoost‐SHAP, ridge regression and KNN	Tumor delivery and organ‐specific biodistribution	Retrospective in vivo–derived datasets; mostly internal validation	Promising but endpoint‐dependent; prospective external validation remains limited.
Therapeutic candidate prioritization	Munteanu et al. [94]; Li et al. [106]	2	PTML/bagging and ML‐guided chemical‐library screening	Antiglioblastoma activity and mRNA transfection potency	Li 2024 included prospective synthesis and in vivo testing	Strongest evidence where AI‐selected candidates were prospectively tested.
Nanotoxicity prediction	Ahmadi et al. [101]; Khokhlov et al. [105]	2	Classification/regression models	Toxicity class, viability, and dynamic toxicity	Compiled in vitro/in vivo toxicity records	Relevant to nanoparticle safety; inclusion depends on whether the protocol permits safety‐only studies.
Deep‐learning LNP outcome prediction	Harrison et al. [93]	1	Deep‐learning image models	Early prediction of delivery/expression outcomes	Experimental microscopy	Innovative, but reporting and external validation remain limited.

*Note:* A meta‐analysis is not appropriate because the studies used heterogeneous nanoparticle systems, datasets, endpoints, algorithms, and performance metrics.

### 3.4. Toxicity Prediction and Safety Assessment

Safety evaluation is a key problem to address while creating delivery systems based on nanoparticles. There were two studies where different machine‐learning techniques were utilized to predict the danger of nanoparticles through the data on physicochemical properties and the levels of exposure received by them in practice [101, 105]. The research made by Ahmadi et al. included several studies and tried one machine‐learning method, which helped in classifying nanoparticles based on physicochemical properties and the levels of exposure [101]. At the same time, Khokhlov et al. introduced their own machine‐learning model, which predicted changes in the toxicity of nanoparticles based on certain parameters and duration of exposure [105].

All the above‐stated studies acknowledge the significance of physicochemical properties in the assessment of the toxicity of nanoparticles, but they also show how machine learning contributes to estimation processes. Nevertheless, both of the studies depend on the datasets that were derived from internal sources, which does not give a chance to make use of them in practice [101, 105].

This means that learned facts establish that machine learning is a useful instrument in predicting nanotoxicity and could be beneficial in the search for the most suitable formulations of nanoparticles at an early stage of development. The next step for regular implementation of this method in the process of safety assessment is testing it on larger data sets with fixed methods of presenting predictive results.

### 3.5. Future Perspectives and Research Gaps

However, despite the promising applications of AI in the area of nanoparticle formulation optimization, biodistribution prediction, nanotoxicity assessments, and material discovery, most of the studies included in this paper remain preclinical in nature. First, the majority of the conducted studies were based on retrospective datasets, models validated within the study, or experimental proof‐of‐concept studies, with few prospective external validation studies reported. Future research efforts should concentrate on producing standardized datasets, fostering multicenter collaboration, encouraging prospective validation studies, and promoting the transparency of model development and performance in order to enhance reproducibility and applicability of findings [95–107].

## 4. Discussion

This systematic review synthesized and critically evaluated the rapidly expanding applications of AI in the design, optimization, and evaluation of NDDSs. Across 17 included studies, AI demonstrated strong potential to improve formulation efficiency, mechanistic insight, safety prediction, and translational relevance. However, significant methodological, biological, and regulatory limitations remain, particularly for emerging domains such as personalized nanomedicine. Importantly, none of the included studies demonstrated real‐world patient‐level deployment, clinical workflow integration, or prospective validation of personalized AI‐guided nanomedicine systems. Thus, personalization in this field remains exploratory and largely speculative.

Similarly, emerging concepts such as reinforcement learning, graph neural networks, digital twins, multiomic integration, and adaptive dosing are discussed as prospective directions based on the broader literature and were not represented as established applications among the 17 primary studies included in this systematic review.

### 4.1. AI Enables More Efficient and Predictive Nanoparticle Formulation

Several included studies reported improved predictive performance compared with conventional statistical and regression‐based approaches for predicting nanoparticle physicochemical attributes, including particle size, PDI, encapsulation efficiency, and release kinetics. However, these comparisons were generally study‐specific and not directly comparable across investigations because different datasets, prediction tasks, and evaluation metrics were used [95, 97, 98, 102–104].

AI demonstrated considerable potential to improve nanoparticle formulation development by predicting critical quality attributes, including particle size, PDI, encapsulation efficiency, zeta potential, and drug‐release behavior. Across the included studies, machine‐learning algorithms such as ANNs, RFs, XGBoost, ensemble learning, and Bayesian optimization were applied to optimize formulation composition and manufacturing parameters, supporting more efficient and data‐driven formulation development.

Importantly, several included studies reported reductions in the number of experimental iterations required during formulation optimization. In formulation development, AI‐assisted optimization reduced the need for empirical formulation iterations by prioritizing promising formulation candidates and optimizing experimental design before experimental validation. This approach improved development efficiency while reducing reliance on conventional trial‐and‐error formulation strategies [97, 102–104].

Beyond formulation optimization, the included studies demonstrate the expanding role of AI across nanoparticle research. Machine‐learning approaches progressed from the prediction of nanoparticle physicochemical properties to biodistribution modeling, nanotoxicity assessment, nano–bio interaction prediction, and therapeutic material discovery. Tree‐based ensemble methods, particularly RF and XGBoost, were frequently applied for formulation optimization because of their ability to model heterogeneous experimental datasets, whereas deep‐learning approaches were primarily used for image‐based analyses and lipid nanoparticle delivery prediction. AI‐assisted PBPK models further extended these applications to the prediction of nanoparticle biodistribution and tumor delivery [93, 96, 99, 106, 107]. Collectively, these findings demonstrate that AI is evolving from retrospective prediction toward active support of nanoparticle design and development. Nevertheless, most studies relied on retrospective datasets and internal validation, while prospective external validation remained limited. Consequently, larger standardized datasets, multicenter collaborations, and prospective validation studies will be essential to improve the robustness and generalizability of AI‐assisted nanoparticle formulation models [96, 99, 107].

### 4.2. AI‐Based Prediction of Physicochemical Properties and Biological Performance

The biological relevance of AI‐predicted properties underscores the value of computational modeling. Particle size, surface charge, morphology, hydrophilicity, and protein‐corona profiles are key determinants of biodistribution, immune recognition, cellular uptake, and therapeutic index. AI models demonstrated predictive capability for these properties across diverse nanoparticle platforms, strengthening safe‐by‐design strategies [91, 92, 95, 98, 100].

XAI was represented in one included study. Ma et al. combined XGBoost with SHAP to identify the relative contribution of nanoparticle physicochemical properties and tumor genomic characteristics to delivery efficiency. This approach improved model interpretability and demonstrated how XAI can support mechanistic understanding of nanoparticle targeting while maintaining predictive performance [100].

Nonetheless, the predictive performance of AI models depends heavily on the quantity, diversity, and quality of training datasets. Many included studies used small datasets, which increases the risk of overfitting and limits generalizability. Larger, well‐curated datasets—and community efforts to standardize nanoparticle descriptors—are needed to unlock the full potential of AI for biological prediction.

Despite encouraging predictive performance, most studies relied on retrospective datasets and internal validation, highlighting the need for larger standardized datasets and prospective external validation to improve model generalizability [96, 99, 107].

### 4.3. AI‐Driven Biodistribution and PK Modeling Provide Translational Value

AI‐assisted PBPK models and machine‐learning approaches have been applied to predict nanoparticles distribution in the body, accumulation of these particles in tumors, and delivery to targeted organs. Notably, four of the papers assessed focused on biodistribution of nanoparticles, using retrospective experimental information combined with computational tools [96, 99, 100, 107].

Based on a study by Lin et al., a combination of various machine‐learning methods with PBPK modeling allowed for forecasting delivery to tumors at different time intervals, and it was found that predictive power varied according to different biological endpoints and experimental settings [96]. Chou et al. also developed a PBPK approach, applying AI and using the data from 288 studies, and recounted *R* ^2^ being ≥ 0.70 for 133 records. In particular, the data on transfer to tumors amounted to around 0.82–0.83 [99]. Nevertheless, the noted results were obtained just for a bit less than half of the records studied.

Along with PBPK modeling, the authors utilized the XGBoost algorithm combined with SHAP to establish tumor delivery efficiency and determine how different physicochemical properties of nanoparticles and tumor genomic characteristics affect the predictions made by the proposed model [100]. More recently, Mahdi et al. have proposed a model based on Bayesian ridge regression, kernel ridge regression, and K‐nearest neighbor methods to evaluate the nanoparticle delivery efficiency in the various organs and observed that the results obtained differ based on the organ [107].

Together, these results suggest that AI‐aided biodistribution modeling has great promise for improving predictions of nanoparticle transport and tumor localization. However, most of the studies were based on retrospective information and internal validation, while biological validation and independent external validation were considerably lacking in the majority of cases. The present evidence confirms the methodological potential of AI‐aided biodistribution modeling but does not prove its generalizability and clinical applicability [96, 99, 100, 107].

This limitation demonstrates a number of challenges on the path toward practical adoption. In particular, biological validation will require specific experimental facilities, large funding, and cooperation between computational specialists and experimental scientists. Moreover, there are no accepted validation standards for AI‐based nanoparticle modeling, which makes comparing studies difficult and raises questions about their repeatability. Thus, further development will rely on the future collaboration of specialists in the fields of computational and experimental science and on possible development of validation standards.

### 4.4. AI‐Assisted Nanotoxicity Prediction and Safety Assessment

AI has been investigated as an approach for predicting nanoparticle toxicity during early stage nanoparticle development. Two of the studies used machine‐learning techniques to estimate the toxicity of nanoparticles based on their physicochemical characteristics as well as experimental results [101, 105].

Ahmadi et al. evaluated various machine‐learning algorithms for predicting nanoparticle toxicity with the help of already collected databases of nanotoxicity. Their research showed that machine learning can be positively used for classifying nanoparticle toxicity using physicochemical and experimental characteristics [101]. In the same manner, Khokhlov et al. created machine‐learning models for classifying and building a conceptual model of nanoparticle toxicity considering the changes in toxicity at different points in time [105].

While encouraging predictive performance was offered by both studies, the reliance on predominantly retrospective datasets as well as the internal validation of their models resulted in uncertainty regarding their broad applicability over nanoparticle platforms, experimental conditions and biological systems. Moreover, discrepancies between the datasets, modeling strategies and predictive performance metrics further complicate making straightforward comparisons between them.

Considering these facts, the results suggest that AI‐enabled prediction of nanotoxicity could help identify unsafe nanoparticle formulations early on and contribute to the development of safer nanoparticles overall. Yet, it is crucial to conduct further validation studies, create standardized datasets, and report predictive power metrics in a more consistent manner before these approaches could become a standard practice in terms of nanoparticle safety assessments [101, 105].

### 4.5. Future Perspectives and Research Gaps

The studies included in this review illustrate the potential usefulness of AI in the fields of nanoparticle formulation optimization, biodistribution prediction, nanotoxicity evaluation, and therapeutic agent development. However, up to now, the evidence is limited to preclinical applications only. The majority of studies used either retrospective data or internally validated models (or both) as well as proof‐of‐concept experiments. There are hardly any reports of the use of prospective external or clinical validation [96, 99, 107].

The main limitation noted about the studies included in this review is the problem of obtaining a large number of standard samples for neural network development. The differences in nanoparticle systems, and approaches applied, biological endpoints, and type of reporting in published articles complicate the comparison between studies. The possible solution is to develop standard datasets and standardized reporting.

In the future, collaborations among multiple centers and biological validation methods should be prioritized along with standardized reporting of model development performance. It is possible that better comprehension of the models may be achieved through the implementation of techniques such as XAI , experimental validation, and PBPK modeling. Meanwhile, it is necessary to validate the application of AI in nanoparticle design.

### 4.6. Quality Assessment Highlights Methodological Gaps

Supporting Table S1 provides the complete details of quality assessment findings for the studies considered as part of this review. In total, ten studies were identified as high quality, while seven of them were identified as of moderate quality using the modified TRIPOD‐AI and JBI appraisal framework. Notably, none of the studies could be assessed as of low quality. Relevant values, scores, and ratings for the studies are available in Supporting Table S1.

The quality assessment identified several recurring methodological strengths and limitations. Although several studies provided adequate descriptions of their datasets, predictors, analytical methods, and validation procedures, external validation remained limited. Only four of the 17 studies used independent external datasets for validation, whereas most relied primarily on internal validation. Biological validation was also less frequently reported, particularly among studies focused on nanotoxicity. Other methodological limitations included insufficient reporting of data preprocessing, hyperparameter tuning, and calibration methods in some studies. In addition, the predominance of retrospective datasets and limited availability of a consistent benchmark dataset reduced the reproducibility of the results and restricted AI models′ applicability to other nanoparticle platforms and biological systems.

In conclusion, the results give a clear understanding of the role of standardized reporting, prospective external validation, a stronger emphasis on biological validation, and the increased adoption of some reporting frameworks like TRIPOD‐AI in improving methodological quality.

### 4.7. Comparison With Prior Reviews

Previous narrative reviews have highlighted the potential of AI to support nanoparticle drug delivery; however, most primarily summarized emerging technologies without systematically evaluating methodological quality, validation strategies, or the maturity of the available evidence [2, 8, 50, 90, 108, 109].

In contrast, the present systematic review synthesizes evidence from 17 studies using predefined eligibility criteria and incorporates methodological quality assessment together with evaluation of experimental and biological validation where reported.

Compared with earlier reviews, the present study places a greater emphasis on the strengths and limitations of current AI applications across nanoparticle formulation optimization, biodistribution prediction, nanotoxicity assessment, nano–bio interaction modeling, and therapeutic material discovery. In particular, this review highlights the predominance of retrospective datasets, limited external validation, and substantial methodological heterogeneity as key barriers to the broader translation of AI‐assisted nanoparticle development into pharmaceutical research and future clinical applications.

### 4.8. Overall Interpretation

The results of this systematic review show that AI has been increasingly applied across different stages of nanoparticle drug delivery, such as formulation development, physicochemical property prediction, biodistribution modeling, toxicity testing, and discovery of new drugs. All studies included in this review suggest that AI can model complicated relations between the formulation parameters and characteristics of nanoparticles, therefore supporting more data‐driven nanoparticle development.

Despite these advances, most studies remain preclinical. In most studies analyzed in this review, the data were retrospectively obtained and internally validated, while only a few studies performed independent validation. Moreover, a lot of differences were found among the nanoparticles used, datasets created, predictions made, methods used, and metrics obtained. This variety of methodologies made it impossible to use any quantitative synthesis method and indicate that standardizing reporting and validation methods would be very helpful for making similar studies more reproducible and applicable for various situations.

One more noteworthy point is that AI serves mainly as a decision‐making aid rather than as a substitute for experimental research. The studies that were reviewed in the report have shown that computation‐based predictions have been beneficial when combined with experimental confirmation, biological testing, and mechanistic modeling methodologies, including PBPK models. This combined method promises more opportunities for proper nanoparticle design than using either modeling or experimental methods separately.

In general, there are signs that AI could enhance the pace of nanoparticle studies and make formulation development and biological behavior prediction easier. Yet, substantial progress in this direction requires larger and standard sets of data, multi‐institution validation, openness about AI model design, and closer connection with experimental methods used in pharmaceutical and medical practices.

## 5. Conclusion

The field of nanoparticle research, which deals with the formulation of drugs and drug delivery systems, greatly benefits from the application of AI technology with respect to the optimization of formulations, predicting the properties of nanoparticles, modeling biodistribution, assessing nanotoxicity, and discovering therapeutically active compounds. Nevertheless, it can be concluded that the usefulness of AI in nanoparticle research is closely connected with the amount and quality of data on nanoparticles, as well as the level of validation of AI methods used. Further research should focus on the creation of standardized datasets and providing multicenter approaches to developing the needed methods of validation.

## 6. Limitations and Future Directions

First, although a large number of records were identified during the literature search, only 17 studies met the predefined eligibility criteria. This may reflect publication bias because studies reporting positive AI performance are more likely to be published than those reporting neutral or negative findings. Second, substantial methodological heterogeneity across the included studies—including differences in nanoparticle platforms, AI algorithms, prediction targets, validation strategies, datasets, and reported performance metrics—precluded formal quantitative meta‐analysis. Consequently, the evidence was synthesized using a structured narrative approach supported by descriptive summaries of study characteristics and reported performance metrics. Although these descriptive summaries facilitate comparison of broad trends across studies, they do not account for differences in study size, methodological quality, or effect magnitude and therefore should not be interpreted as quantitative evidence of consistency or comparative performance of specific AI approaches. Third, most included studies were preclinical proof‐of‐concept investigations with limited external validation and almost no clinical integration, thereby restricting the generalizability and translational interpretation of the findings.

In the future, more research should focus on the creation of standardized datasets for evaluating nanoparticles, independent external validation studies, consistent descriptions of the creation and functioning of AI models, and greater connection between computational models and practical experiments. Use of frameworks such as TRIPOD‐AI can help achieve better transparency of results. Moreover, collaborating with other teams in several medical institutions and using standardized nanoparticle description and validation will make it easier to assess the reliability of AI models in the future.

In conclusion, these limitations highlight the necessity of standard datasets, rigorous validation, transparent reporting, and more extensive collaboration between computation and experimentation to ensure the future application of nanoparticle drug delivery systems based on AI.

## Funding

This research was funded by Jadara University.

## Conflicts of Interest

The author declares no conflicts of interest.

## Supporting Information

Additional supporting information can be found online in the Supporting Information section.

## Supporting information


**Supporting Information** The complete literature search strategy for this systematic review is shown in Appendix A, containing the databases, Boolean search strings and limits, eligibility criteria, and selection process based on the PRISMA 2020 recommendations. Table S1 provides the methodological assessment of the 17 included studies based on the modified TRIPOD‐AI and its JBI framework. Table S2 provides the operational definitions and scoring criteria applied in the evaluation of methodological quality.

## Data Availability

Data sharing is not applicable to this article as no datasets were generated or analyzed during the current study.
