# Supplementary material for: Artificial Intelligence in the Design and Development of Nanoparticle Drug Delivery Systems: A Systematic Review
Source: Adv Pharmacol Pharm Sci. 2026 Sep 21;2026:5531134. doi: 10.1155/adpp/5531134 (PMC13591434; doi:10.1155/adpp/5531134)
Supplement: Supplementary file 1 [file ADPP-2026-5531134-s001.zip › Supplementary File 1.docx]

# Appendix A: Database Search Strategy and Rationale

This appendix provides the complete database search strategy used for the systematic review titled “Artificial Intelligence in the Design and Development of Nanoparticle Delivery Systems: A Systematic Review.” The search followed PRISMA 2020 guidelines to ensure comprehensiveness, reproducibility, and transparency.

## Databases Searched

The following electronic databases were systematically searched:
• PubMed/MEDLINE
• Scopus
• Web of Science (Core Collection)
• IEEE Xplore
• ScienceDirect
• Google Scholar

## Search Timeframe

Publications from January 2015 to January 2025 were included, covering a decade of progress in AI applications for nanomedicine and drug delivery.

Following peer-review, all eligible studies were re-audited against the original publications to verify eligibility, bibliographic accuracy, study characteristics, and reported outcomes. This verification resulted in refinement of the final evidence base while preserving the original search strategy and eligibility criteria.

## Search Terms and Boolean Combinations

Controlled vocabulary (e.g., MeSH terms) and free-text keywords were combined using Boolean operators (AND/OR). A representative search string used in Scopus and Web of Science was:

("artificial intelligence" OR "machine learning" OR "deep learning" OR "neural networks" OR "reinforcement learning") AND ("nanoparticle" OR "nanoformulation" OR "nanocarrier" OR "nanomedicine" OR "nanotechnology") AND ("drug delivery" OR "targeted delivery" OR "formulation optimization" OR "controlled release")

## Search Rationale

Search terms were chosen to capture a broad spectrum of AI-driven modeling, prediction, and optimization strategies for nanoparticle systems. Database selection ensured representation of biomedical, pharmaceutical, and computational disciplines. IEEE Xplore captured AI algorithmic and engineering studies, PubMed/MEDLINE and Scopus retrieved biomedical and pharmaceutical research, and Google Scholar included grey literature, theses, and preprints.

## Search Limits and Filters

Filters were applied to restrict results to English-language publications, peer-reviewed journals, and full-text availability. Duplicates were removed using EndNote 20 before screening.

## Reproducibility

All search records were exported and archived. Two independent reviewers validated each search to ensure accuracy and completeness. The final list of included studies is provided in Table 1 of the manuscript.
