# Supplementary material for: Artificial Intelligence in the Design and Development of Nanoparticle Drug Delivery Systems: A Systematic Review
Source: Adv Pharmacol Pharm Sci. 2026 Sep 21;2026:5531134. doi: 10.1155/adpp/5531134 (PMC13591434; doi:10.1155/adpp/5531134)
Supplement: Supplementary file 1 [file ADPP-2026-5531134-s001.zip › Supplementary Tables.docx]

**Supplementary Table S1:** Methodological Quality Assessment of the Included Studies Using the Modified TRIPOD-AI and JBI Framework

| **Study** | **Dataset Description** | **Model Transparency** | **Predictor Selection** | **Internal Validation** | **External Validation** | **Biological Validation** | **Calibration / Performance Reporting** | **Outcome Measurement** | **Statistical Methods** | **Total Score** | **Quality** |
| --- | --- | --- | --- | --- | --- | --- | --- | --- | --- | --- | --- |
| **Ban et al. (2020)** | Yes | Yes | Yes | Yes | Partial | Yes | Yes | Yes | Yes | 13.5 | High |
| **Yan et al. (2020)** | Yes | Partial | Yes | Yes | No | Yes | Partial | Yes | Yes | 11.0 | Moderate |
| **Harrison et al. (2021)** | Yes | Yes | Yes | Yes | No | Yes | Yes | Yes | Yes | 12.0 | High |
| **Munteanu et al. (2021)** | Yes | Yes | Yes | Yes | No | No | Yes | Yes | Yes | 11.0 | Moderate |
| **Wang et al. (2022)** | Yes | Yes | Yes | Yes | No | Yes | Yes | Yes | Yes | 12.0 | High |
| **Lin et al. (2022)** | Yes | Yes | Yes | Yes | Partial | No | Yes | Yes | Yes | 12.5 | High |
| **Dawoud et al. (2023)** | Yes | Yes | Yes | Yes | No | Yes | Yes | Yes | Yes | 12.0 | High |
| **Hoseini et al. (2023)** | Yes | Yes | Yes | Yes | No | No | Yes | Yes | Yes | 11.0 | Moderate |
| **Chou et al. (2023)** | Yes | Yes | Yes | Yes | Yes | Partial | Yes | Yes | Yes | 13.5 | High |
| **Ma et al. (2023)** | Yes | Yes | Yes | Yes | No | No | Yes | Yes | Yes | 11.0 | Moderate |
| **Ahmadi et al. (2024)** | Yes | Yes | Yes | Yes | No | No | Yes | Yes | Yes | 11.0 | Moderate |
| **Chaurawal et al. (2024)** | Yes | Yes | Yes | Yes | No | Yes | Yes | Yes | Yes | 12.0 | High |
| **Seegobin et al. (2024)** | Yes | Yes | Yes | Yes | No | Yes | Yes | Yes | Yes | 12.0 | High |
| **Maharjan et al. (2024)** | Yes | Yes | Yes | Yes | Partial | Yes | Yes | Yes | Yes | 13.0 | High |
| **Khokhlov et al. (2024)** | Yes | Yes | Yes | Yes | No | No | Yes | Yes | Yes | 11.0 | Moderate |
| **Li et al. (2024)** | Yes | Yes | Yes | Yes | Partial | Yes | Yes | Yes | Yes | 13.0 | High |
| **Mahdi et al. (2025)** | Yes | Yes | Yes | Yes | Partial | No | Yes | Yes | Yes | 12.5 | High |

**Supplementary Table S2:** Operational definitions and scoring criteria used for methodological quality assessment of included studies based on the modified TRIPOD-AI and Joanna Briggs Institute (JBI) appraisal framework.

| **Domain** | **Yes (1)** | **Partial (0.5)** | **No (0)** |
| --- | --- | --- | --- |
| **Dataset description** | Complete description of dataset source, size, and characteristics | Some dataset information missing | Not reported |
| **Model transparency** | AI model architecture, workflow, and implementation clearly described | Partially described | Insufficient description |
| **Predictor selection** | Predictor variables clearly defined and justified | Predictor variables partially described | Not reported |
| **Internal validation** | Appropriate cross-validation and/or independent training–test split reported | Limited validation procedures | No internal validation |
| **External validation** | Model evaluated using an independent external dataset | Limited external assessment | No external validation |
| **Biological validation** | Experimental validation using in vitro and/or in vivo studies | Limited experimental validation | No biological validation |
| **Calibration / performance reporting** | Appropriate performance metrics (e.g., R², RMSE, MAE, AUROC, accuracy) clearly reported | Performance metrics incompletely reported | Performance metrics not reported |
| **Outcome measurement validity** | Outcome measures clearly defined and appropriate | Minor limitations | Poorly defined outcomes |
| **Statistical methods** | Statistical analyses appropriately described and justified | Partially described | Not reported |
